# Actin turnover maintains actin filament homeostasis during cytokinetic ring contraction

**DOI:** 10.1083/jcb.201701104

**Published:** 2017-09-04

**Authors:** Ting Gang Chew, Junqi Huang, Saravanan Palani, Ruth Sommese, Anton Kamnev, Tomoyuki Hatano, Ying Gu, Snezhana Oliferenko, Sivaraj Sivaramakrishnan, Mohan K. Balasubramanian

**Affiliations:** 1 Warwick Medical School, University of Warwick, Coventry, UK; 2 Key Laboratory of Regenerative Medicine, Ministry of Education, Jinan University, Guangzhou, China; 3 Department of Genetics, Cell Biology, and Development, University of Minnesota, Minneapolis, MN; 4 Randall Division of Cell and Molecular Biophysics, King’s College London, London, UK; 5 Francis Crick Institute, London, UK

## Abstract

Cytokinesis in many eukaryotes involves a tension-generating actomyosin-based contractile ring. Many components of actomyosin rings turn over during contraction, although the significance of this turnover has remained enigmatic. Here, using *Schizosaccharomyces japonicus*, we investigate the role of turnover of actin and myosin II in its contraction. Actomyosin ring components self-organize into ∼1-µm-spaced clusters instead of undergoing full-ring contraction in the absence of continuous actin polymerization. This effect is reversed when actin filaments are stabilized. We tested the idea that the function of turnover is to ensure actin filament homeostasis in a synthetic system, in which we abolished turnover by fixing rings in cell ghosts with formaldehyde. We found that these rings contracted fully upon exogenous addition of a vertebrate myosin. We conclude that actin turnover is required to maintain actin filament homeostasis during ring contraction and that the requirement for turnover can be bypassed if homeostasis is achieved artificially.

## Abstract (precis)

Many cytokinetic actomyosin ring components undergo dynamic turnover, but its function is unclear. Chew et al. show that continuous actin polymerization ensures crucial F-actin homeostasis during ring contraction, without which ring proteins organize into noncontractile clusters.

## Introduction

An actomyosin-based contractile machinery containing F-actin, myosin II, and actin cross-linkers is instrumental in tension generation in diverse cellular processes (Murrell et al., 2015). During cytokinesis, most eukaryotic cells assemble a dynamic actomyosin ring that contracts to drive cleavage furrow ingression (Pollard and Wu, 2010). The cytokinetic actomyosin ring is dynamic, and several ring components, such as F-actin, myosin II, and actin-associated proteins, exchange with the cytosolic pool of these proteins to varying degrees during contraction (Yumura, 2001; Pelham and Chang, 2002; Guha et al., 2005; Murthy and Wadsworth, 2005; Mukhina et al., 2007; Clifford et al., 2008; Goldbach et al., 2010; Uehara et al., 2010; Calvert et al., 2011; Wloka et al., 2013; Srivastava and Robinson, 2015). In this article, we define this exchange and replenishment process as turnover. Theoretical studies of actomyosin ring contraction suggest a requirement of actin turnover in supporting myosin II–dependent contraction (Stachowiak et al., 2014; Oelz et al., 2015; Oelz and Mogilner, 2016). In addition, turnover of actin filaments has been proposed to generate contractile stress independently of myosin II (Zumdieck et al., 2007; Mendes Pinto et al., 2012). Although experimental results that are consistent with a role for turnover in actomyosin ring contraction exist (Pelham and Chang, 2002; Silva et al., 2016), experiments that test this hypothesis are scarce. Thus, the functional significance of turnover of actin and other ring proteins remains unknown.

Here, using the fission yeast *Schizosaccharomyces japonicus*, we investigate this question in cells and spheroplasts, as well as in isolated intact and fixed actomyosin rings in cell ghosts. *S. japonicus* was used for this work because it divides using an actomyosin ring, is well suited for imaging because of its large size, is readily permeable to cytoskeletal inhibitors, and has aspects of cytokinesis that are related to those in animal cells (Gu et al., 2015).

## Results and discussion

In studies of actomyosin ring contraction in *S. japonicus* using FRAP, we observed a dynamic turnover of many ring components including Rlc1 (myosin II regulatory light chain), Cdc15 (F-BAR protein), Myo2 and Myp2 (myosin II heavy chains), and Rng2 (IQGAP; Fig. S1 A). To address the significance of this turnover of Rlc1-GFP, we investigated contraction of Rlc1-GFP–expressing rings in cell ghosts devoid of cytoplasm and cell wall (Young et al., 2010; Mishra et al., 2013; Huang et al., 2016a; Fig. S1, B and C).

**Figure 1.**
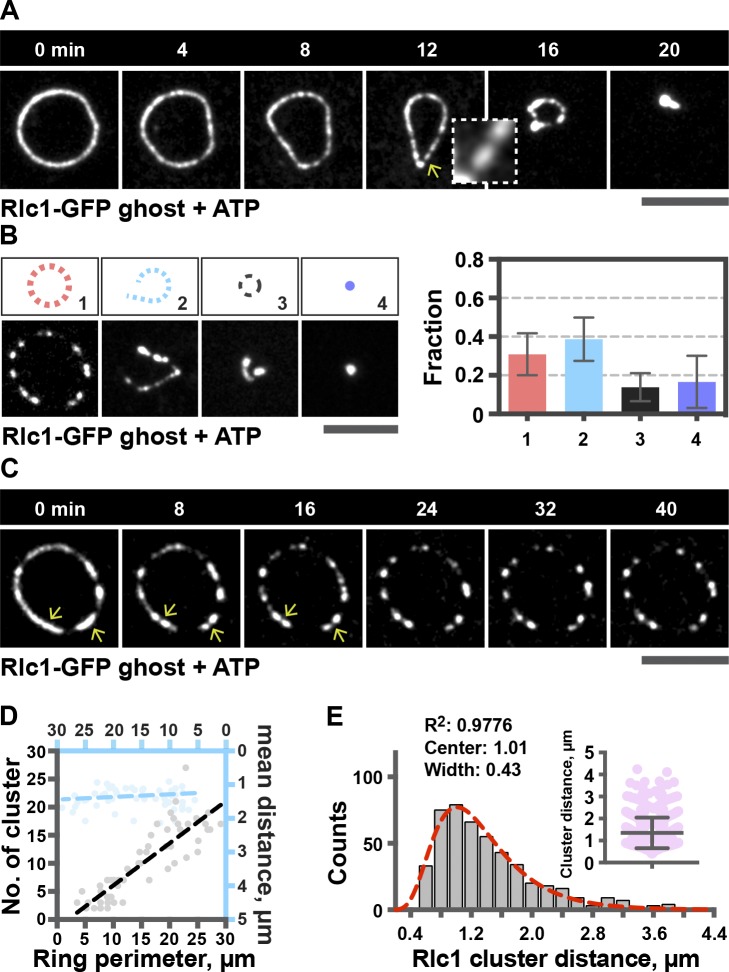
**Formation of clusters containing actomyosin ring components in the absence of turnover.** (A) Contraction of rings in cell ghosts incubated with 0.5 mM ATP. Arrow, clusters. (B) Different categories of rings in cell ghosts incubated with 0.5 mM ATP. Shown are representative images of each category after 50-min ATP incubation. 1, 70 rings; 2, 88 rings; 3, 33 rings; 4, 37 rings. (C) Formation of Rlc1 clusters in rings in cell ghosts incubated with 0.5 mM ATP. Arrows, clusters. (D) Quantification of the number of clusters as a function of ring perimeter (black dotted line; 53 rings), and quantification of the mean distance between clusters as a function of ring perimeter (blue dotted line; 50 rings). The ring perimeter quantitated is the initial ring perimeter after ATP addition. (E) Distribution of the distance between Rlc1 clusters (478 clusters). Red line, log-Gaussian fitted line to the distribution; *R*^2^, fitting coefficient. All the distances between clusters are plotted in the scattered dot plot.

Upon incubation of cell ghosts with ATP, some of the rings contracted fully without any observable membrane invagination (Figs. 1 A and S1 D). As anticipated in a system without cytosol, FRAP experiments failed to detect appreciable recovery of Rlc1-GFP fluorescence, in the presence or absence of ATP (Fig. S1 E). Interestingly, in ATP-treated cell ghosts, Rlc1-GFP signal was frequently distributed unevenly and tended to form clusters (Fig. 1 A). We found that ring contraction profiles could be classified into four categories (Fig. 1 B): (1) clustering with no significant contraction (30.9 ± 10.8%); (2) clustering with ring breakage during contraction (38.6 ± 11.2%); (3) incomplete contraction (13.9 ± 7.3%); and (4) full contraction (16.6 ± 13.5%). Even in rings that underwent contraction, myosin II was distributed in a nonhomogeneous manner, although this was not as prominent as in rings that failed to contract (Fig. 1, compare A and cell ghost 1 in B). These experiments revealed that ring contraction in the absence of cytosol and cell wall was an inefficient process, with only ∼17% of rings undergoing full contraction. In the majority of rings in cell ghosts, Rlc1-GFP formed clusters upon ATP addition, and these rings failed to contract further (Fig. 1 C and Video 1). It appeared that the number of clusters formed during ring contraction scaled proportionally with the ring perimeter (Fig. 1 D; Pearson *R*^2^ = 0.7441), with clusters distributed 1.35 ± 0.69 µm apart (Fig. 1 E). The mean distance between clusters, however, did not scale with ring perimeter (Fig. 1 D; Pearson *R*^2^ = 5.625 × 10^−5^). A similar distribution of myosin II in regularly spaced clusters has been reported previously (Stachowiak et al., 2012; Alvarado et al., 2013; Mak et al., 2016). It has been proposed that the cluster periodicity may be related to myosin II–dependent actin reorganization, during which myosin II localizes to regions of zero actin polarity (Stachowiak et al., 2012), possibly driven by network connectivity (Alvarado et al., 2013; Mak et al., 2016).

Myo2, Myp2, Cdc15, Rng2, and Cdc12 formed clusters upon ATP addition (Fig. S1, F and G) in addition to Rlc1. Some of the Rlc1 clusters partially colocalized with Cdc12 clusters (Fig. S1 G). These experiments established that in the absence of cytosol and cell wall, *S. japonicus* actomyosin ring proteins tend to form uniformly spaced clusters, leading to inefficient contraction. Although actomyosin rings in *Schizosaccharomyces pombe* cell ghosts undergo ATP-dependent contraction (Mishra et al., 2013), in our quantitative experiments, we found that ∼63% of actomyosin rings contracted fully, whereas rings in ∼37% of cell ghosts reorganized into clusters, as in *S. japonicus* ghosts (Fig. S1 H).

**Figure 2.**
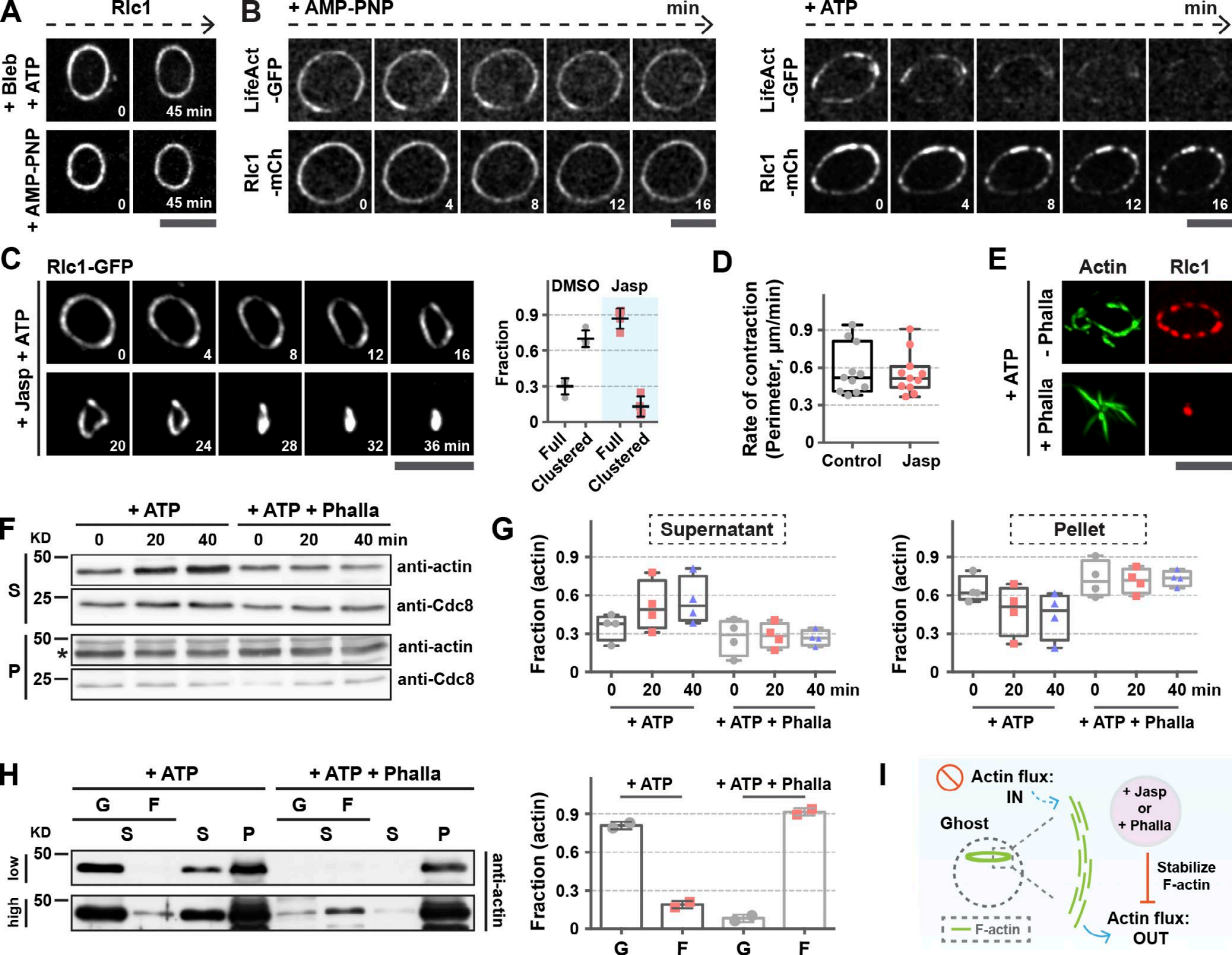
**The majority of rings in cell ghosts undergo full contraction upon stabilization of actin filaments.** (A, top) Rlc1-GFP rings in cell ghosts incubated with 0.5 mM ATP and 100 µM blebbistatin. (Bottom) Rlc1-mCherry rings in cell ghosts incubated with 0.5 mM AMP-PNP. (B) Rlc1-mCherry rings in cell ghosts were stained with GST-LifeAct-GFP and incubated with 0.5 mM AMP-PNP (four rings) or 0.5 mM ATP (11 rings). (C) Contraction of rings in cell ghosts in the presence of 20 µM jasp (56 rings); rings in cell ghosts that underwent full-ring contraction versus those that formed clusters were quantitated. Full, full-ring contraction. (D) The change of Rlc1-GFP ring perimeters over time in cell ghosts was quantitated (11 rings each sample). (E) Rlc1-mCherry rings in cell ghosts were incubated with ATP with or without 5 µM Pha for 40 min, and then stained with purified GST-LifeAct-GFP. (F) Pha treatment stabilizes actin filaments in rings in cell ghosts. Proteins were extracted from ATP-treated rings in cell ghosts with or without Pha, and immunoblots were probed with α-actin or Cdc8p. Asterisks, actin; S, supernatant; P, pellet. (G) Quantification of the intensity of bands in protein blots (four protein blots). (H) An ultracentrifugation assay of the actin proteins during contraction of rings in cell ghosts. G, globular actin; F, filamentous actin; S, supernatant; P, pellet. Quantification of the band intensity on the protein blots (two protein blots). Shown at top and bottom are blots exposed for different durations. The intensity of bands (G and F lanes) in protein blots was quantitated. (I) Inhibition of loss of actin filaments (flux out) by jasp or Pha from the rings in cell ghosts improves ring contraction efficiency in the absence of actin polymerization (flux in).

Previous work has shown that the amount of F-actin in the ring decreases during contraction (Kamasaki et al., 2007; Mishra et al., 2013) and that myosin II can break and release actin filaments within networks (Guha et al., 2005; Murthy and Wadsworth, 2005; Murrell and Gardel, 2012; Vogel et al., 2013). We therefore hypothesized that clustering could be the result of myosin II–dependent actin filament disassembly, leading to myosin II accumulation at the remaining actin foci. Consistently, cluster formation was almost fully abolished upon incubation of cell ghosts with the myosin II inhibitor blebbistatin and ATP or with the nonhydrolyzable ATP analog AMP-PNP (Fig. 2 A). As expected, these rings did not contract.

Next, we tested whether fluorescence intensity of actin filaments was reduced in rings in cell ghosts incubated with ATP. Actin intensity did not reduce appreciably in ghosts treated with LifeAct-GFP and AMP-PNP (Fig. 2 B, +AMP-PNP). However, actin intensity in cell ghosts treated with ATP and LifeAct-GFP reduced over time, although the Rlc1-GFP intensity remained unaffected (Fig. 2 B, +ATP; Fig. S2 A; and Video 2). We therefore investigated whether stabilizing actin filaments in rings in cell ghosts with drugs prevented clustering of myosin and reversed the ring contraction defect. We treated cell ghosts with the actin-stabilizing drug jasplakinolide (jasp) and found that the number of rings in cell ghosts that underwent full contraction increased significantly (Fig. 2 C and Video 3). 87.5% of rings in cell ghosts contracted fully upon jasp treatment, compared with 23.6% of control rings that contracted fully (Fig. 2, C [graph] and D [contraction rates]). Similarly improved ring contraction was observed when cell ghosts were incubated with the actin-stabilizing drug phallacidin (Pha; Fig. S2 B, graph). Although the rings in cell ghosts treated with Pha completed contraction with intact actin filaments attached to the contracted Rlc1 structure, the actin filaments were disorganized, and Rlc1 formed clusters in untreated cell ghosts (Fig. 2 E). Other actin modulators did not prevent myosin clustering (Fig. S2 B). That actin stabilization increased efficiency of contraction and prevented clustering suggests that clustering of ring components was caused by defective turnover and was unrelated to the lack of cell wall.

The cell ghosts accumulated more G-actin and Cdc8 tropomyosin in supernatants over time after ATP addition, indicating that more actin and associated proteins were released from rings in cell ghosts during contraction (Fig. 2, F and G). Contraction of rings in cell ghosts in the presence of Pha, however, resulted in the retention of more actin and Cdc8 in pellets (Fig. 2, F and G). Ultracentrifugation analysis of the supernatant of cell ghosts after ATP addition revealed that the actin was largely filamentous when contraction of rings in cell ghosts was triggered in the presence of Pha (Figs. 2 H and S2 D). Collectively, these experiments revealed that in the absence of turnover, ring components tended to cluster potentially because of a reduction in the amount of ring-associated F-actin. Because incubation of rings with actin stabilizers reversed clustering of ring proteins and improved the efficiency of ring contraction, we hypothesized that continuous actin polymerization helped replenish the lost actin filaments (Fig. 2 I). We tested this idea by asking whether rings that had been transformed into clusters in the presence of ATP (referred to as clustered rings) could reinitiate contraction upon actin polymerization by addition of G-actin. Clustered rings were incubated with an actin polymerization mixture containing Cdc12(FH1FH2) and Cdc3 profilin and ATP, with or without G-actin. Clustered rings held in the absence of G-actin remained static, whereas those rings held in the presence of G-actin underwent minor contraction (perimeter shrank ∼2 µm in 60 min), with clusters undergoing movements (Fig. S2 C and Video 4), consistent with the idea that continuous actin polymerization helped replenish lost actin filaments.

**Figure 3.**
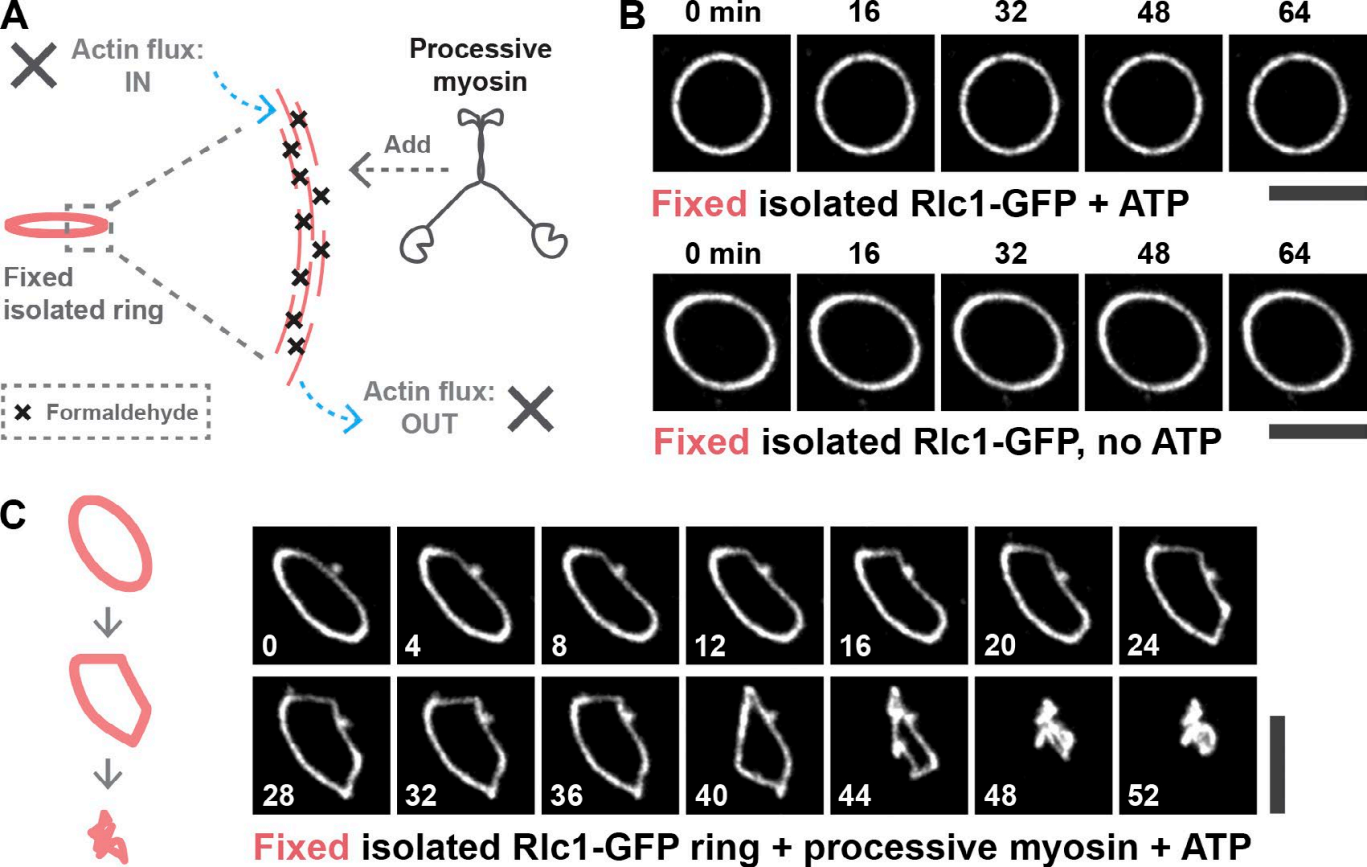
**Processive vertebrate myosin-dependent contraction of formaldehyde-fixed *S. japonicus* actomyosin rings.** (A) Schematic representation of the experimental design. (B) Formaldehyde-fixed rings incubated with ATP do not contract (top). Addition of a processive myosin does not induce ring contraction without ATP (bottom). (C) Contraction of the fixed rings incubated with a processive myosin and ATP (nine rings).

We next used an independent approach to test our hypothesis that actin filament homeostasis, rather than continuous actin polymerization, was important for proper ring contraction. To completely abolish actin turnover during ring contraction, we cross-linked the rings in cell ghosts by fixation with formaldehyde (Fig. 3 A). This method cross-links actin networks while preserving the domain on actin responsible for its interaction with myosin (Tee et al., 2015). After fixing with formaldehyde, the rings in cell ghosts did not contract upon ATP addition, suggesting that the native myosin II complexes present in the rings had been inactivated by formaldehyde treatment (Fig. 3 B). Next, we tested whether contraction of the formaldehyde-fixed rings could be achieved by addition of an exogenous myosin motor. We chose a version of a type V myosin motor, which is highly processive, taking >10 steps each of ∼40 nm (Hariadi et al., 2014). The exogenously added myosin V, remarkably, promoted contraction (leading to a contortion caused by a lack of actin network disassembly or expulsion of actin bundles) of the fixed rings upon addition of ATP (Fig. 3 C and Video 5; *n* = 9 rings). Contraction and contortion of the fixed rings in ghosts by processive myosin is consistent with the idea that ring contraction itself does not require a dynamic turnover of actin filaments, provided a sufficient density of actin filaments is available.

**Figure 4.**
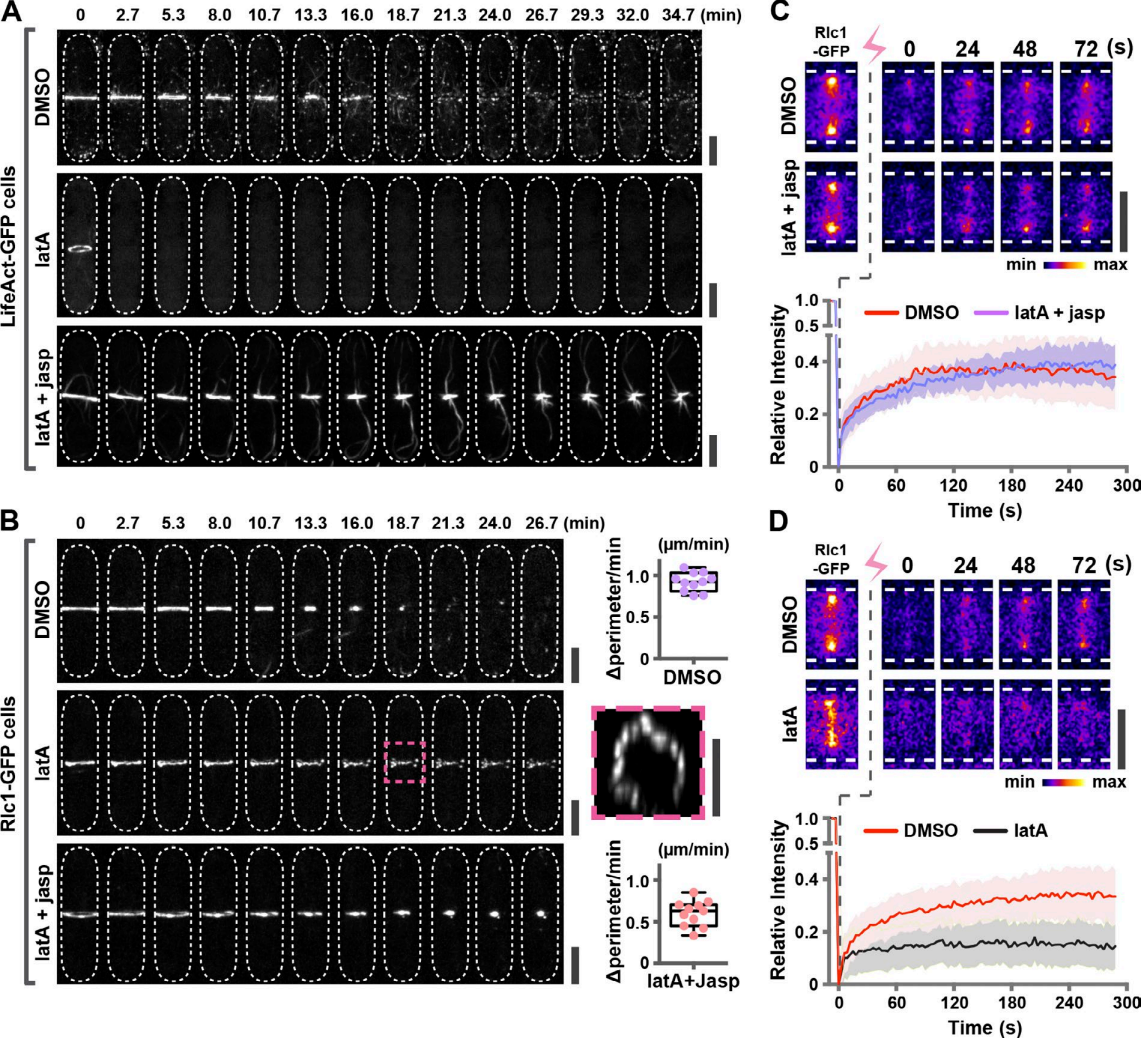
**Actin filament homeostasis supports ring contraction in cells.** (A) *S. japonicus* cells expressing LifeAct-GFP were incubated with 100 µM latA (middle; 20 cells) or 100 µM latA and 100 µM jasp (bottom; 24 cells). DMSO was used as a control (top; 21 cells). (B) *S. japonicus* cells expressing Rlc1-GFP were incubated with 100 µM latA (middle; 24 cells) or 100 µM latA and 100 µM jasp (bottom; 30 cells). DMSO was used as a control (top; 21 cells). The rate of contraction (perimeter changes over time) was quantitated for cells incubated in DMSO (11 cells) or latA and jasp (11 cells). Box, 3D rotation of the ring at time point 18.7 min. (C) FRAP of Rlc1-GFP on the rings treated with latA and jasp. DMSO, seven cells; latA + jasp, 10 cells. (D) FRAP of Rlc1-GFP on the rings treated with latA alone in less than 10 min. DMSO, 10 cells; latA, 13 cells.

We further tested this idea in a cellular context. First, we perturbed the input of actin molecules into the rings by treating cells expressing LifeAct-GFP or Rlc1-GFP with latrunculin A (latA; Fig. 4, A and B; and Videos 6 and 7). As expected, F-actin was lost upon latA treatment, and Rlc1 reorganized into clusters, whereas treatment with DMSO (solvent) did not affect F-actin or Rlc1 behavior. Importantly, treatment of cells with latA and jasp to block actin turnover (Peng et al., 2011; Hu et al., 2017) prevented Rlc1 clustering and allowed ring contraction, although the rate of ring contraction was slower than in DMSO-treated cells (Fig. 4, A and B; and Videos 6 and 7). When actin turnover was blocked and actin filament homeostasis was achieved, Rlc1-GFP displayed a dynamic turnover in FRAP experiments as in DMSO-treated cells, although Rlc1-GFP turnover was abolished in latA-treated cells (Fig. 4, C and D).

**Figure 5.**
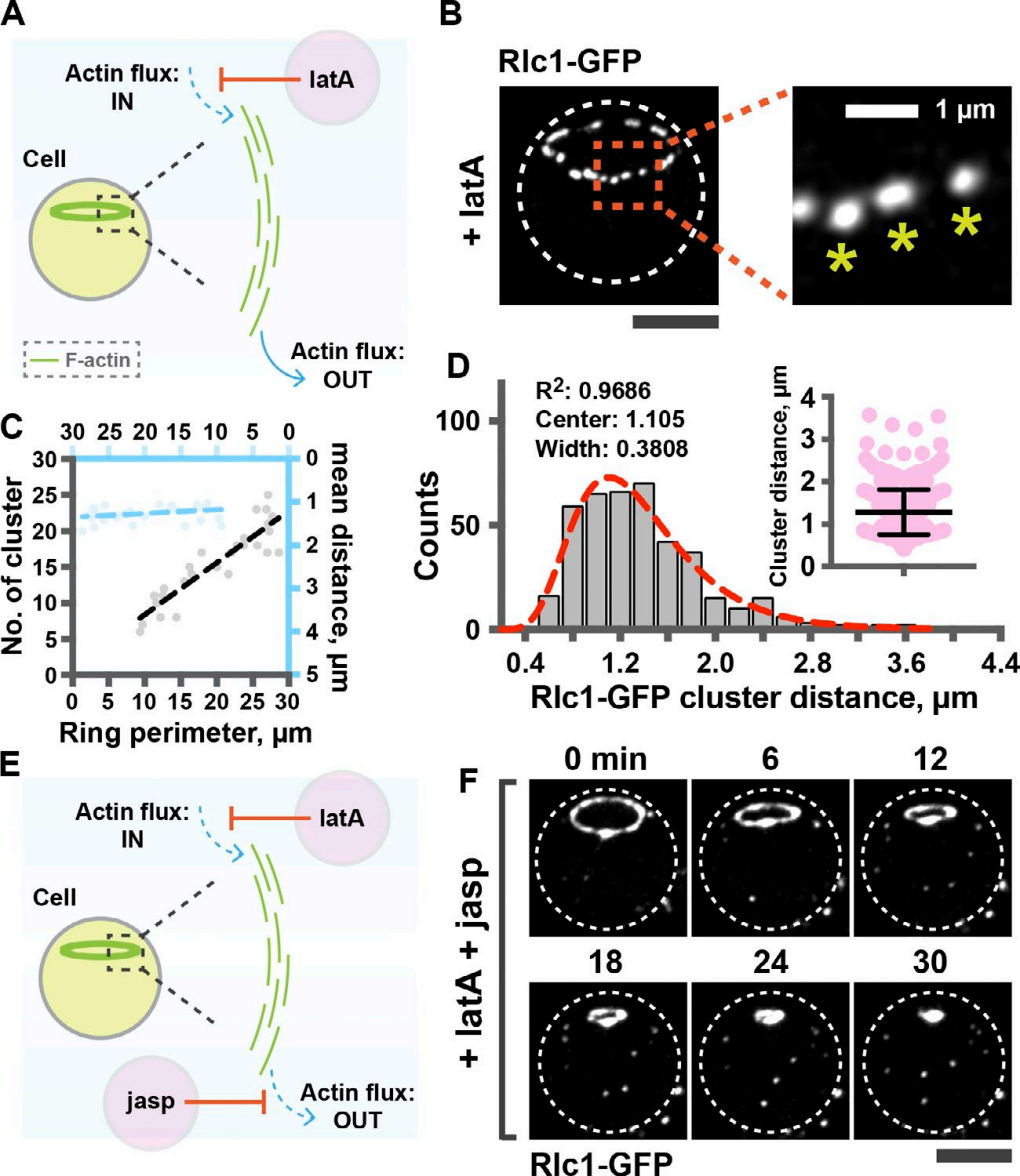
**Actin filament homeostasis supports ring contraction in spheroplasts.** (A) Schematic representation of the experimental design. (B) Formation of Rlc1-GFP clusters upon 100 µM latA treatment (10 rings). Asterisks, clusters. (C) Quantification of the number of clusters as a function of ring perimeter (27 rings), and quantification of the mean distance between clusters as a function of ring perimeter (27 rings). (D) Distribution of the distance between clusters (410 clusters). Red line, log-Gaussian fitted line to the distribution; *R*^2^, fitting coefficient. The scattered dot plot shows all the distances between clusters. (E) Diagram illustrating the perturbation of actin turnover in spheroplasts by using latA and jasp. (F) Contraction of rings in spheroplasts after 100 µM latA and 100 µM jasp treatment (12 rings).

We next chose to study ring contraction in spheroplasts so as to remove potential complicating contributions from cell wall assembly in ring contraction. Also, spheroplasts aided in easy visualization of rings in the axial plane. As before, we perturbed the input of actin molecules into the rings by treating spheroplasts expressing Rlc1-GFP with latA (Fig. 5 A). After treatment with latA for 15 min, when nearly all actin filaments were lost (Fig. S3 A), Rlc1-GFP molecules on the rings formed distinct clusters (Fig. 5 B). Similarly, in latA-treated spheroplasts, Myo2, Cdc15, Rng2, and Cdc12 formed clusters (Fig. S3 B) whose morphology resembled those generated during contraction of rings in cell ghosts. The number of myosin clusters after latA treatment scaled proportionally to the initial ring perimeter (Fig. 5 C; Pearson *R*^2^ = 0.8191), and the distance between myosin clusters was 1.28 ± 0.53 µm in both larger and smaller rings (Fig. 5, C and D; Pearson *R*^2^ = 0.04654). Thus, absence of continuous actin polymerization led to formation of clusters containing myosin II and other ring components in spheroplasts.

As seen in cells (Fig. 4, A and B), treatment of spheroplasts with latA and jasp prevented Rlc1 clustering and allowed ring contraction at a rate of ∼0.31 µm/min (Fig. 5, E and F; Fig S3 C; and Video 8). Collectively, our work supports the idea that actin filament network homeostasis is sufficient to prevent clustering of ring proteins on the actomyosin ring and to support proper contraction (Fig. S3 D).

In summary, we have shown that myosin II and other actomyosin ring proteins form clusters in cell ghosts that lack cytosol and G-actin and in latA-treated cells and spheroplasts. Recent work has shown that cytokinetic node proteins are observed in distinct clusters during ring contraction in *S. pombe*. Because Mid1, which is key to node organization, was not investigated during contraction (Laplante et al., 2016), more work will be required to clarify the relationship between clusters we have described and the Mid1-dependent nodes. That myosin clusters are uniformly spaced, and that clustering can be prevented by actin stabilizers, indicates that clustering is a result of self-organization of ring components in the absence of turnover, and not the uneven attachment of the actomyosin ring to plasma membrane or cell wall. A similar clustering has been observed upon stimulation of myosin II on actin networks in vitro (Soares e Silva et al., 2011; Stachowiak et al., 2012). It is possible that the clusters represent the end points of cytokinetic “contractile units” described previously (Carvalho et al., 2009; Silva et al., 2016).

Our work is consistent with the notion that the actomyosin network is intrinsically unstable and prone to collapse into clusters in the absence of actin turnover, instead of undergoing full contraction (Stachowiak et al., 2014; Oelz et al., 2015; Mak et al., 2016). Interestingly, formation of clusters is abolished when rings in ghosts are treated with blebbistatin, suggesting a role for myosin II in the cluster formation of ring proteins. This suggests that actin filaments are likely broken or released through buckling by myosin II (Murrell and Gardel, 2012; Vogel et al., 2013), leading to a nonhomogeneous distribution of myosin II, which associates with the remaining actin filaments.

The formation of clusters can be suppressed and full-ring contraction can be restored by treatment of rings with actin-stabilizing drugs, even when actin polymerization is inhibited. It is likely that continuous actin polymerization repairs actin tracks that are damaged or removed during contraction, ensuring actin filament homeostasis. The presence of actin filaments of mixed polarity generated through polymerization (Kamasaki et al., 2007) might enable roughly homogeneous redistribution of myosin II (Wollrab et al., 2016) and other ring proteins and suppression of cluster formation, causing continuous symmetric contraction. Thus, maintenance of actin filament homeostasis by actin turnover coupled with myosin processivity (Miyazaki et al., 2015; Oelz et al., 2015) and connected disordered actin networks (Alvarado et al., 2013; Stachowiak et al., 2014; Oelz et al., 2015; Ennomani et al., 2016) collectively ensure proper actomyosin ring contractility.

In a formaldehyde-fixed actomyosin ring system, we found that rings contract without actin polymerization or disassembly. Similarly, actomyosin rings in *S. pombe* cell ghosts or synthetic vesicles contract in the absence of actin dynamics (Mishra et al., 2013; Miyazaki et al., 2015). This establishes that actin disassembly or polymerization themselves are not required for ring contraction, but that these are the consequences and responses to ring contraction. Our findings also explain seemingly inconsistent experiments describing sensitivity or resistance to the actin sequestering compound latA during cytokinetic ring contraction (Pelham and Chang, 2002; Carvalho et al., 2009). It is possible that different organisms use different strategies to ensure actin filament homeostasis.

## Materials and methods

### Yeast strains, medium, and culture conditions

*S. japonicus* strains used are listed in Table S1. Strains were prepared as previously described (Gu et al., 2015). Cells were cultured in rich medium YEA (5 g/l yeast extract, 30 g/l glucose, and 225 mg/l adenine) until midlog phase at 24°C for physiological analysis. Spheroplasts were prepared by enzymatic digestion with lytic enzymes Lallzyme MMX (16207; Lallemand; Flor-Parra et al., 2014) and recovered in YEA medium containing sorbitol until the actomyosin rings were assembled. LatA (BML-T119; Enzo Life Sciences) and jasp (ALX-350-275; Enzo Life Sciences) were used at the final concentration of 100 µM each to perturb the actin dynamics in cells and spheroplasts. SMIFH2 (#344092; EMD Millipore), CK-666 (SML0006; Sigma-Aldrich), phallacidin (ab143532; water soluble; Abcam), blebbistatin (B0560; Sigma-Aldrich), and AMP-PNP (A2647; Sigma-Aldrich) were used at final concentrations as specified in the figure legends. FM4-64 (T3166; 1 mg/ml; Thermo Fisher Scientific) was used at the final concentration of 0.625 µg/ml.

### *S. japonicus* spheroplast and cell ghost preparation and ATP-dependent contraction of rings in cell ghosts

*S. japonicus* cell ghosts were prepared by published methods (Huang et al., 2016a,b). In brief, a rich YEA medium was used to culture the *S. japonicus* cells until midlog phase, and lytic enzymes Lallzyme MMX (Lallemand; Flor-Parra et al., 2014) was used to digest the cell wall for spheroplast preparation. The resulting spheroplasts were washed and recovered in sorbitol-containing medium. After the actomyosin rings were assembled, spheroplasts were washed once with wash buffer (20 mM Pipes-NaOH, pH 7.0, 0.8 M sorbitol, 2 mM EGTA, and 5 mM MgCl_2_) and permeabilized with isolation buffer (50 mM Pipes-NaOH, pH 7.0, 0.16 M sucrose, 50 mM EGTA, 5 mM MgCl_2_, and 50 mM potassium acetate) containing 0.5% NP-40 to obtain cell ghosts. After permeabilization, cell ghosts were washed twice with reactivation buffer (0.16 M sucrose, 5 mM MgCl_2_, 50 mM potassium acetate, and 20 mM MOPS-NaOH, pH 7.0; pH adjusted to 7.5). The isolation buffer and reactivation buffer were chilled to 4**°**C, and the isolation and washing steps were performed on ice. To induce ATP-dependent actomyosin ring contraction, equal volumes of cell ghosts and reactivation buffer containing 1 mM ATP (A6559; Sigma-Aldrich) were mixed. The time 0 frames indicated in the figures for cell ghost imaging are the beginning of the time-lapse imaging. Thus, these frames are at least 1–2 min after addition of ATP, which is the time it takes to mount the ghosts and find ghosts appropriate for imaging.

### *S. pombe* cell ghost preparation

The *S. pombe* cell ghosts were prepared as previously described (Mishra et al., 2013; Huang et al., 2016b). The *S. pombe* culture at the midlog growth phase was spun down at 3,000 rpm for 1 min and washed once with an equal volume of E-buffer (50 mM sodium citrate and 100 mM sodium phosphate, pH 6.0). The cells were spun down and resuspended in 10 ml E-buffer containing 1.2 M sorbitol. The cell suspension was incubated with 30 mg lysing enzyme (L1412; Sigma-Aldrich) at 24°C with shaking at 80 rpm for 90 min, followed by continuous incubation with 20 µl LongLife Zymolyase (1.5 U/µl; G-Biosciences) at 24°C with shaking at 80 rpm for 60 min. The cells after enzymatic digestion were spun down at 450 *g* for 2 min and washed once with 5 ml E-buffer containing 0.6 M sorbitol. The spheroplasts were then spun down at 450 *g* for 2 min and recovered in 10 ml minimal medium containing 0.8 M sorbitol and full supplements until a large number of spheroplasts assembled actomyosin rings. After the actomyosin rings were assembled, spheroplasts were washed once with wash buffer and permeabilized with isolation buffer containing 0.5% NP-40 to obtain cell ghosts. After permeabilization, cell ghosts were washed twice with reactivation buffer. The isolation buffer and reactivation buffer were chilled to 4**°**C, and the isolation and washing steps were performed on ice. The cell ghosts were stored in reactivation buffer containing 20% glycerol. The rings in cell ghosts were then immobilized and perfused with reactivation buffer containing 0.5 mM ATP using a CellASIC ONIX Microfluidic system (Merck Millipore).

### Drug treatments in spheroplasts and cells

To treat cells with latA in Fig. 4 (A and B), 25 µl cells were incubated with a premix containing 0.5 µl of 5 mM latA and 0.5 µl DMSO and imaged immediately. For the treatment of latA and jasp in Fig. 4 (A and B), 25 µl cells were incubated with 0.5 µl of 5 mM jasp for 1–2 min and mixed with 0.5 µl of 5 mM latA before imaging.

For latA treatment in Figs. 5 B and S3 A, a premix containing 0.5 µl of 5 mM latA and 0.5 µl DMSO was added to 25 µl spheroplasts, and the treated spheroplast suspension was imaged. For the treatment of latA and jasp in Fig. 5 F, 0.5 µl of 5 mM latA was premixed with 0.5 µl of 5 mM jasp, and 25 µl spheroplasts were added to this premix for imaging.

### Sample preparation for microscopy imaging

To image cells or spheroplasts in suspensions, 1 ml yeast cell culture at midlog growth phase or spheroplasts (regenerated in YEA medium containing 1 M sorbitol for 1.5–3 h) was spun down at 450 *g* for 2 min, and 16 µl concentrated samples were imaged in an Ibidi µ-Slide eight-well glass-bottom dish (#80827). To image ATP-dependent contraction of rings in cell ghosts, an equal volume of reactivation buffer containing 1 mM ATP was added to cell ghosts and imaged in an Ibidi µ-Slide eight-well glass-bottom dish. All imaging dishes were sealed with an adhesive film membrane to prevent water evaporation during imaging.

To image the formaldehyde-fixed rings, the rings in cell ghosts were fixed with 3.7% formaldehyde for 12 min at RT and washed three times with reactivation buffer. Before imaging, the fixed rings were further washed once with motility buffer (20 mM imidazole, pH 7.5, 25 mM KCl, 4 mM MgCl_2_, 1 mM EGTA, and 10 mM DTT) and resuspended in the same buffer. To induce fixed ring contraction, 1 µl of 8 µM calmodulin, 1 µl Cy3-labeled hybrid myosin V/VI (carrying myosin V head with myosin VI lever arm; Hariadi et al., 2014), and 1 µl of 100 mM ATP were mixed with 13 µl fixed rings in cell ghosts. Imaging was done at RT.

To image clustered rings in Fig. S2 C, the Rlc1-GFP rings in cell ghosts were preincubated with 0.5 mM ATP for 2–3 h to induce cluster formation. The clustered rings in cell ghosts were then incubated with 114 µg/ml GST-Cdc12(FH1FH2), and mixed with 2.5 µM human β-actin and 9 µM Cdc3 profilin in the reactivation buffer containing 0.5 mM ATP. For the control experiments, the clustered rings in cell ghosts were incubated with 114 µg/ml GST-Cdc12(FH1FH2) and mixed with 0.1 mg/ml BSA and 9 µM Cdc3 profilin in the reactivation buffer containing 0.5 mM ATP.

### Time-lapse imaging and quantification of F-actin in cell ghosts

To visualize F-actin in cell ghosts in Figs. 2 B and S2 A, 8 µl rings in cell ghosts were incubated with 1 µl of 0.0325 µg/µl recombinant GST-LifeAct-GFP protein (freshly diluted from the stock of 1.3 µg/µl each time before imaging) for 1–2 min at RT. The rings in cell ghosts were further mixed with 0.5 µl of 5 mg/ml BSA and 8 µl of 1 mM AMP-PNP or ATP before imaging.

To quantitate the fluorescence intensity of LifeAct-GFP and Rlc1-mCherry in ATP treatment (Fig. S2 A), the total fluorescence intensity of each tagged protein was obtained by performing SUM projections of raw image stacks along the *z*-axis (Fiji/image/stacks/z project). Next, the actomyosin rings were manually outlined in each time frame using line region of interest (ROI) of 1-µm line width. The raw intensity (I_raw_) was derived as sum of all pixel values in ROI at each time frame. In parallel, the area of each ROI (ROI_area_) was measured and recorded.

To calculate the background intensity in each time series, one ROI (4 × 4 µm) away from ring structures by at least 2 µm was selected from the first, middle, and last time frames. The background intensity (I_background_) was calculated as mean intensity per pixel in the selected ROI.

To estimate the loss of fluorescence intensity caused by photobleaching during image acquisition, we used AMP-PNP–treated rings in cell ghosts as the control samples. The control samples were imaged in conditions identical to the ATP-treated samples. The total fluorescence intensity of each tagged protein was obtained by performing SUM projections of raw image stacks along the *z*-axis (Fiji/image/stacks/z project). Next, the actomyosin rings were manually outlined in each time frame using line ROI of 1 µm line width. The raw intensity was then derived as sum of all pixel values in ROI in each time frame. The sum intensity was imported into custom Matlab scripts to quantitate the loss of fluorescence. To generate the bleaching profile for each ring, the total fluorescence intensity at each time frame was normalized to the fluorescence intensity of the first time frame. The means of the normalized values at each time frame were calculated and used to fit a two-term exponential function *f*(*t*) with Matlab function fit(exp2).

For each ring at each time frame, the corrected intensity of each tagged protein (I_corrected_) for time frame *t* was calculated as I_raw_(*t*) × 1/*f*(*t*). The intensity of the tagged-proteins (I_protein_) was calculated as I_corrected_(*t*) – [I_background_ × ROI_Area_] and then normalized to the first time frame. The mean and SD of the normalized values at each time frame were calculated and plotted.

### F-actin staining

To visualize F-actin structures in Fig. 2 E, rings in cell ghosts after different treatments were fixed with 3.7% formaldehyde for 12 min at RT and washed twice with reactivation buffer. The recombinant GST-LifeAct-GFP protein (1.3 µg/µl) was added at 1:100 into the fixed cell ghosts and incubated overnight at 4°C. The cell ghosts were washed twice with reactivation buffer before imaging.

### Spinning-disk confocal microscopy

Andor Revolution XD spinning disk confocal microscopy was used for the image acquisition. The microscope was equipped with a Nikon Eclipse Ti inverted microscope, Nikon Plan Apo Lambda 100×/1.45-NA oil-immersion objective lens, a spinning-disk system (CSU-X1; Yokogawa Electric Corporation), and an Andor iXon Ultra EMCCD camera. Images were acquired using the Andor IQ3 software at 80 nm/pixel. The fluorophores were excited by laser lines at wavelengths of 488 or 561 nm. Most images were acquired with *z*-step sizes of 0.2, 0.3, or 0.5 µm, at various interval times indicated in individual time-lapse videos.

### FRAP

FRAP experiments in Fig. S1 A (Cdc15-GFP, mNG-Myo2, Myp2-mNG, and GFP-Rng2) and Fig. S1 E were done using a spinning disk confocal microscope (Andor Revolution XD). FRAP experiments in Fig. 4 (C and D) and Fig. S1 A (Rlc1-GFP) were done using a laser scanning confocal microscope (Zeiss LSM710 or LSM880).

The Zeiss LSM710/880 was equipped with a 100× (oil-immersion, 1.4-NA, apochromat) or 60× (oil-immersion, 1.4-NA, plan apochromat) objective lens. Image acquisition and hardware were controlled by Zen software (ZEISS). To detect Rlc1-GFP molecules, GFP fluorophores were excited at 488 nm (argon laser), and the emitted light was collected by a PMT array in the range of ∼495 to 600 nm. The detector pinhole was open to 10 Airy units to improve the signal. Frame size was adjusted to achieve resolution of 90 nm/pixel in the final images.

The spinning disk confocal system used in FRAP experiments was equipped with a Nikon ECLIPSE Ti inverted microscope, Nikon Plan Apo Lambda 100×/1.45-NA oil-immersion objective lens, a spinning-disk system (CSU-X1; Yokogawa Electric Corporation), an Andor iXon Ultra EMCCD camera, and Andor FRAPPA unit. Images were acquired using the Andor IQ3 software at 69 nm/pixel. The fluorophores were excited by laser lines at wavelengths of 488 nm.

The fluorophores were photobleached by using both 405- and 488-nm laser lines (laser scanning confocal) or a 445-nm line (spinning disk confocal). A bleaching ROI was drawn either as a 2 × 4-µm rectangle engulfing the ring (laser scanning confocal) or a ∼2–4-µm line ROI along the ring plane (spinning disk confocal). The conditions for bleaching duration and laser power were adjusted to achieve maximum bleaching efficiency of the ring with minimal depletion of the cytoplasmic pool (∼5–20% in this study). Three frames of prebleached images were acquired before photobleaching, followed immediately by time-lapse acquisition of images. Bleaching was completed in a mean of ∼50–200 ms depending on number of bleaching ROIs. For FRAP recovery curve quantification, a single *z*-slice at the middle plane of the cell was acquired every 3 s for a total duration of 5 min.

The fluorescence intensity of FRAP images was quantitated using Fiji. The mean intensity was calculated for each time point for a rectangular ROI containing the ring. The mean intensity of the Rlc1-GFP signal in cytoplasm of cells not subjected to bleaching was measured for the bleaching correction. The measured data in CSV format were loaded into custom-written Matlab scripts.

For the Matlab scripts, every individual measurement (bleached and postbleach intensities) was normalized to a mean value of the first three time points (prebleach intensity). Next, the mean value and SD were calculated for each time point for respective groups (bleaching control, DMSO-treated, or drug-treated cells). To compensate for bleaching during imaging, a two-term exponential curve was fitted (*a* × exp(*b* × *x*) + *c* × exp(*d* × *x*); function fitted with exp2 model) to mean values of the time-lapse data in bleaching control. After fitting, bleaching correction value *C* was calculated for each time point as 1/fit(*t*). Corrected FRAP value was then recalculated as mean(*t*) × *C*. Next, the fourth-time point (bleached images) for each FRAP curve was set to 0 to subtract inputs from background and cytoplasmic diffusion. The final plot was built as mean value per time point per group with SD of each point.

### Image analysis and processing

Images were analyzed using Fiji and Imaris (Bitplane). The image stacks were projected along the *z*-axis using either sum-intensity or maximum-intensity projections for analysis and representation. The sum-intensity projections were performed in Fig. S2 A for quantification. The maximum-intensity projections were used in the remaining micrographs. Background subtraction (Fiji/process/subtract background) was performed in all microscopy images except those for fluorescence intensity quantification. All time-lapse microscopy images except micrographs in Figs. 2 B, 4 (A, C, and D), S1 (A and H), and S2 C were corrected for photobleaching (Fiji/image/adjust/bleach correction).

Imaris was used to facilitate 3D measurements in Fig. 1 (D and E) and Fig. 5 (C and D). The distance between two neighboring aggregates in these figures was quantitated by the shortest path between the centers of two aggregates.

All time-lapse videos were edited and saved in MP4 format with H.264 compression using Fiji and Adobe Photoshop. Adobe Illustrator was used to arrange figures. Graphs were made with Prism 6.0 (GraphPad Software). Scale bars in all figures are 5 µm, unless stated otherwise. Error bars represent SD.

### Preparation of recombinant myosin

Recombinant myosin was prepared as previously described (Hariadi et al., 2015). The construct contained (N to C terminus): the motor domain of myosin Va (residues 1–815; *Gallus gallus*), the lever arm of myosin VI (residues 917–992; *Sus scrofa*), a GCN4 leucine zipper (for dimerization), a SNAP tag (for Cy3 labeling), and a FLAG tag (for purification). Myosin was cloned in pBiex-1 (71234; EMD Millipore) and expressed through transient transfection in Sf9 insect cells using the Escort IV system (L3287; Sigma-Aldrich). Cells were lysed, and clarified lysate was incubated with anti-FLAG resin (Sigma-Aldrich) for 1 h at 4°C. Myosin bound to resin was then labeled with Cy3 DNA oligonucleotides overnight, washed, and eluted with FLAG peptides (Sigma-Aldrich). Labeled myosin was stored at −20°C in 20 mM imidazole, 150 mM KCl, 5 mM MgCl_2_, 1 mM EDTA, 1 mM EGTA, 1 mM DTT, 55% (vol/vol) glycerol, 1 µg/ml PMSF, 10 µg/ml aprotinin, and 10 µg/ml leupeptin, pH 7.4.

### Preparation of recombinant proteins

Recombinant GST-LifeAct-GFP was expressed in *Escherichia coli* BL21 (DE3-pLysS; Promega) using 0.5 mM IPTG for induction at 30°C for 4 h. The protein was purified on glutathione Sepharose 4B beads according to the manufacturer’s instructions (GE Healthcare). The elution buffer containing glutathione was exchanged with reactivation buffer (0.16 M sucrose, 5 mM MgCl_2_, 50 mM potassium acetate, and 20 mM MOPS-NaOH, pH 7.0; pH adjusted to 7.5) using PD Minitrap G-10 columns (GE Healthcare). The purified proteins were stored at −80°C. Recombinant GST-Cdc3 and GST-Cdc12(FH1FH2) were expressed in *E. coli* and purified. The GST-tag of GST-Cdc3 was cleaved off with Factor Xa protease, and the GST tag was removed by incubating with GSH-Sepharose. Proteins were dialyzed against G-buffer (5 mM Hepes, pH 7.4, 0.2 mM CaCl_2_, 0.01% [wt/vol] NaN_3_, and 0.5 mM DTT) without ATP and stored at −80°C. The human β-actin cDNA with His-Tag at the C terminus was cloned into pPICZc (Invitrogen) and expressed in *Pichia pastoris*. The recombinant protein was purified using a Ni-NTA column, the His-Tag was cleaved off using chymotrypsin, and the protein was stored in G-buffer containing 0.2 mM ATP.

### Immunoblotting and actin sedimentation assay

Samples used in the immunoblots in Fig. 2 (F and H) were prepared as follows: cell ghost pellet and supernatant were separated by centrifugation (15,000 *g*, 5 min at 4°C) after ATP addition (final concentration 0.5 mM) for 20 and 40 min. For the sedimentation assay, three-quarters of the supernatant was subjected to ultracentrifugation at 156,000 *g* for 40 min at 4°C. The soluble fraction after ultracentrifugation contained G-actin or short actin filaments, and the insoluble fraction after ultracentrifugation contained F-actin.

One milliliter of TCA precipitation buffer (250 mM NaOH and 7.5% TCA) was added to resuspend the cell ghost pellet and supernatant, and the suspension was incubated on ice for 10 min. Samples were centrifuged at 17,000 *g* for 25 min at 4°C. Precipitated proteins were resuspended in HU-DTT (200 mM Tris-HCl, pH 6.8, 8 M urea, 5% SDS, 0.1 mM EDTA, 0.005% bromophenol blue, and 15 mg/ml DTT). Samples were heated for 5 min at 95°C before loading on SDS-PAGE gels. Antibodies used were goat anti-actin (gift from J. Cooper, Washington University School of Medicine, St. Louis, MO) and rabbit anti-Cdc8p (Balasubramanian et al., 1992). Secondary antibodies used were rabbit anti–goat and goat anti–rabbit IgGs coupled to HRP (Jackson ImmunoResearch Laboratories, Inc.).

### Quantification of protein intensities

The signal intensities of actin bands on immunoblots in Fig. 2 (F and H) were measured using Fiji. Signal intensities were corrected against the gel background signal. The band intensity of the supernatant (S) and pellet (P) lanes for actin was measured separately and summed. To quantitate the fraction, the band intensity of supernatant lanes was divided by the sum of S and P (fraction = band intensity/sum; sum = S + P). Four measurements were taken from blots derived from four independent experiments in Fig. 2 G. To quantitate the fraction of actin after ultracentrifugation in Fig. 2 H, the band intensity of each fraction was divided by the sum intensity of both G-actin and F-actin fractions. Measurements in Figs. 2 H and S2 D were obtained from two protein blots derived from two independent experiments.

### Statistical analysis

Calculations of mean and SD were done using Prism 6.0.

### Online supplemental material

Fig. S1 shows turnover of actomyosin rings in *S. japonicus* cells and the behavior of cytokinetic ring components in the cell ghost system of *S. japonicus* and *S. pombe*. Fig. S2 shows the quantification of actin intensity, the effects of different compounds on ring contraction in cell ghosts, effects of adding actin and actin nucleators to clustered rings in cell ghosts, and the supernatant/pellet fraction distributions of actin in the biochemical assays. Fig. S3 shows latA treatments of *S. japonicus* spheroplasts expressing different GFP-tagged proteins, the rate of ring contraction in spheroplasts in the presence of latA and jasp, and a graphical abstract of our study. Video 1 (Fig. 1 C) shows formation of Rlc1-GFP clusters in a cell ghost after 0.5 mM ATP treatment. Video 2 (Fig. 2 B) shows disassembly of actin filaments in rings in cell ghosts in the presence of 0.5 mM ATP. Video 3 (Fig. 2 C) shows full contraction of an isolated ring after 0.5 mM ATP and 20 µM jasp treatments. Video 4 (Fig. S2 C) shows cluster movements and minor contraction of clustered rings in cell ghosts in the presence of actin, actin nucleators, and 0.5 mM ATP. Video 5 (Fig. 3 C) shows contraction of a formaldehyde-fixed ring by exogenous myosin V in the presence of ATP. Video 6 (Fig. 4 A) shows the effects of pharmacological treatments of actomyosin rings in cells expressing LifeAct-GFP. Video 7 (Fig. 4 B) shows the effects of pharmacological treatments of actomyosin rings in cells expressing Rlc1-GFP. Video 8 (Fig. 5 F) shows sliding of the Rlc1-GFP ring in a spheroplast after latA and jasp treatments. Table S1 lists yeast strains used in this study.
